# Mulch Films Manufactured from Poly(Butylene Adipate-Co-Terephthalate) and Biopolymers Obtained from Urban and Agriculture Wastes: Mechanical Properties and Effects in Agriculture

**DOI:** 10.3390/polym18121550

**Published:** 2026-06-22

**Authors:** Enzo Montoneri, Philippe Evon, Jordane Charbonnier, Emanuele La Bella, Ferdinando Fragalà, Ivana Puglisi, Andrea Baglieri, Laurent Labonne, Landry Jégat, Solal Mendez, Simone Solaro, Elio Padoan, Jose L. Diéguez

**Affiliations:** 1 Dipartimento di Scienze Agrarie, Forestali e Alimentari, Università Di Torino, 10095 Grugliasco, Italy; elio.padoan@unito.it; 2 Laboratoire de Chimie Agro-Industrielle, Université de Toulouse, INRAE, ENSIACET, 31030 Toulouse, France; philippe.evon@toulouse-inp.fr (P.E.); laurent.labonne@toulouse-inp.fr (L.L.); landry.jegat@toulouse-inp.fr (L.J.); solal.mendez@orange.fr (S.M.); 3 Société D’Extrusion Du Polyéthylène a. Barbier, 43600 Sainte-Sigolène, France; jordane.charbonnier@barbiergroup.com; 4 Dipartimento di Agricoltura, Alimentazione e Ambiente, Università di Catania, Via S. Sofia 98, 95123 Catania, Italyferdinandofragala@virgilio.it (F.F.); ipuglisi@unict.it (I.P.); abaglie@unict.it (A.B.); 5 Hysytech Srl, Via I° Maggio 5, 10043 Orbassano, Italy; simone.solaro@hysytech.com; 6 Simetría Innovación, Calle Filipinas, 39, 46006 Valencia, Spain; jldieguez@simetriagrupo.com

**Keywords:** biopolymers, biowastes, poly(butylene adipate-co-terephthalate), mulch films, mechanical properties, waste derived biopolymers, synthetic biodegradable polymers, biodegradation, deterioration

## Abstract

Biopolymers (BPs), obtained from urban and agricultural wastes, are known as active principles to manufacture ready-for-use finished products in several sectors of the agriculture and chemical industries. These findings prospect a biowaste-based refinery producing chemical specialities to replace products derived from fossil feedstock. The present paper reports new materials containing BPs. Composite granules containing Poly(Butylene Adipate-Co-Terephthalate (PBAT) as a matrix and BPs as fillers are manufactured by twin-screw extrusion. The granules are used to make single-layer PBAT-BP mulch films by single-screw extrusion and three-layer Starch-PBAT-BP films by blown co-extrusion. The films are tested for mechanical properties, and for structural stability and effects in the in vitro cress germination and the in-field horticulture. The results show that both the films’ effects on plant performance and the films’ structural degradation are regulated by the BP and polymeric matrix release kinetics in the operational germination medium or the field soil, and in turn, that the kinetics depend on the mulch film structural features. The horticulture trials prove that the three-layer mulch films have adequate mechanical strength (25 MPa maximum tensile strength and 520% elongation at break) and about 6 months lifespan to maintain and/or improve the soil protection and crop production (17 t/ha) over the plant seasonal cycle. These findings widen the range of renewable chemical specialities potentially producible by the envisioned biowaste-based refinery.

## 1. Introduction

Previous work reports urban and agriculture wastes as potential feedstock for the manufacture of new bio-based polymers (BPs) with multiple purposes for use in diversified sectors of agriculture and the chemical industry [1]. These products are obtained by chemical hydrolysis of fermented unsorted urban or agriculture biowastes. They are mixes of molecules with molecular weights ranging from 5 to over 750 kDa, constituted by aliphatic and aromatic carbon types substituted by acid and basic functional groups, that are in turn bonded to mineral elements of the 1st through the 4th group—memories of the organo-mineral lignocellulosic structure present in the pristine biowastes. As such, BPs have a range of properties that make them multipurpose products for use in several sectors of agriculture and the chemical industry. Results obtained at TRL 3–5, carried out in the laboratory and in a real operational environment, have confirmed that BPs can perform as soil fertilisers, plant growth bio-stimulants and protective agents, biosurfactants for the manufacture of finished consumer products, biopolymers to make plastic articles, catalysts for a variety of chemical and biochemical reactions, auxiliaries for the remediation of polluted dismissed industrial sites, and animal feed supplements. These findings have encouraged the perspective to realise a biowaste-based refinery producing value-added chemical specialities to use in place of the commercial products derived from fossil feedstock.

The chemical and structural features of BP macromolecules represent the memory of the structural chemical features of the fats, polysaccharides, proteins and lignin components in the parent food and agriculture biowastes. The hydrophilic acid and basic functional groups inherited from the fats, polysaccharides, or proteins contribute to the water solubility properties and the performance of BPs as good soil fertilisers and plant growth biostimulants, and as high-performance biosurfactants to perform as active principles in the formulation of finished consumers’ products. However, the inherited lignin aromatic structure represents a drawback for manufacturing plastic articles out of neat BPs. The biopolymers are thermally stable up to 200 °C, but do not melt and have no film-forming properties. Nevertheless, their thermal stability allows fabricating, by solvent casting and melt extrusion, composite articles containing BPs in blends with synthetic polymers such as polyethylene-co-vinyl alcohol (EVOH).

BP blends have been proven to have higher mechanical resistance compared to the article manufactured from the neat synthetic polymer only. BPs are not biodegradable, but they are biocompatible. Based on their sourcing material, no adverse effect is expected from their accumulation in soil. Indeed, the anaerobic digestates and/or compost of municipal biowastes are used as soil amending agents and/or fertilisers. BPs have also been proven more efficient soil fertilisers and plant growth biostimulants in the cultivation of several food and ornamental plants, in comparison with those sourced from fermented urban or agriculture biowastes, and with commercial organo-inorganic peat and leonardite derived products claimed by vendors as plant biostimulants. BPs therefore seemed potential processable fillers, capable of increasing the mechanical resistance of the synthetic polymer matrix in the blend, and performing at the same time as active soil fertilizer and/or plant growth biostimulant active principles in controlled-release fertiliser (CRF) materials.

Here, the authors of the present paper report the manufacture, and the mechanical and CRF properties of new composite poly(butylene adipate-co-terephthalate) (PBAT)-BP mulch films. Several papers are available in the literature that report the production and properties of single-layer and multi-layer films for application in agronomic mulching technology [2]. PBAT-starch three-layer mulch films are a typical example [3]. Consequently, in the present work, new single-layer PBAT-BP and three-layer Starch-PBAT-BP films were both manufactured and then investigated for their properties. This required addressing a number of issues related to the physico-chemical compatibility of BPs with the other film components, and to their effects on the film’s mechanical properties, and their impact in agriculture.

The compatibility of components is the first requirement for blends to guarantee performance in most applications, as with the mulch films in the present case. The choice of PBAT, rather than EVOH polymer matrix, for the manufacture of CRF films was based on several considerations related to the mechanical properties and chemical nature of the two polymers. Firstly, PBAT is a much more flexible and ductile polymer than EVOH. PBAT has a Young’s modulus of 136 MPa [4], compared to around 2850 MPa for EVOH [5]. In parallel, elongation at break is very high for PBAT, being 710% instead of less than 15% for EVOH. Secondly, based on their aromatic C chemical composition, BPs are expected to be more affine and compatible with PBAT than with EVOH.

Compatibilization in polymer blends [6] is necessary due to the different chemical natures of the components. Incompatible components yield heterogeneous mixtures, where the components belong to two different phases. In these cases, the problem is circumvented by adding a specific third component performing as a compatibilizer [7]. This component is expected to have chemical affinity with the two other ones, and so to be able to reduce the interfacial tension between the two phases and to enhance the domain adhesion. In essence, the compatibilizer performs as a surfactant, which improves the miscibility of aqueous and oily phases, and the compatibility between the hydrophobic matrix and hydrophilic filler.

In previous studies [8,9,10], BP blends with EVOH and polyethylene-co-acrylic acid (PEAA) were manufactured using FORSUD and BMP_Z_ BPs, respectively obtained by hydrolysis of the anaerobic digestate of unsorted urban food wastes and of post-harvest tomato plants. In these blends, no third compatibilizer component was added. Compatibilization between the synthetic EVOH and PEAA polymer matrices and the BPs was expected to occur based on the ability of the components to participate in chemical reactions, H-bonding, electron donor–acceptor interactions or Van der Waals bonds between the organic moieties and the functional groups present in the blend components. Indeed, based on the number of hydrophilic and lipophilic moieties, and on their solubility and properties as surfactants, and as emulsifiers, the BPs were expected to be compatible with several synthetic polymers. Therefore, the BPs were used to manufacture processable polymer blends, first by solvent casting and then by melt extrusion [8,9,10]. Compared to the above blends, the PBAT-BP blends reported in the present work were expected to be even more compatible, since both the BP fillers and the PBAT contain also aromatic and carboxyl carbon together with aliphatic C chains.

The reason for manufacturing PBAT-starch three-layer mulch films is that the starch bio-component has been reported to enhance the biodegradability of synthetic PBAT polymer [3]. Plastic biodegradability is often mistakenly considered to be inherently environmentally benign. This oversimplification ignores that some biodegradable formulations can still contribute to pollution, greenhouse gas emissions or land use change [11]. These concerns are particularly pertinent in agriculture, where plastic mulching is widely used to enhance crop productivity. Biodegradable mulch films have emerged as a promising alternative as they offer comparable agronomic performance to polyethylene mulches and can biodegrade directly in agricultural soils under environmental conditions [12]. However, biodegradability alone does not guarantee environmental safety [13].

European standards require biodegradable mulches to comply with strict criteria regarding heavy metals, potentially toxic substances and ecotoxicological effects on living organisms prior to certification [14]. Ecotoxicity is often considered primarily in relation to the biodegradation process itself, based on assumptions derived from composting scenarios where materials are evaluated after disintegration, with intact products presumed to be inert [15,16]. However, biodegradable mulches are deployed on soil surfaces for extended periods, during which they begin to degrade physically and chemically through interactions with environmental factors such as moisture, light, and temperature. Compounds released during this process, including additives, fillers, and intermediate degradation products, may persist in the soil, undergo transformation, or be taken up by plant roots, potentially exerting biological effects [15,16]. Current methods of assessing plant eco-toxicity show limited sensitivity, particularly with respect to root systems and medium- to long-term impacts [17]. Furthermore, studies addressing the eco-toxic potential of biodegradable mulches in soil are scarce, and difficult to compare [17,18,19,20].

In this context, the present work reports the performance of the single-layer PBAT-BP films and of the Starch-PBAT-BP three-layer films, respectively, for the germination of in vitro-cultivated cress seeds, and for the growth of on-field cultivated horticulture plants. The scope of the in vitro and on-field agriculture trials was to attempt a preliminary evaluation of the benefits and criticalities of the new blend films containing BPs, in the outlook of their subsequent industrial and commercial implementation. The major novelty of the present research work is that, notwithstanding the poor plastic properties of the BP connected with its rigid and recalcitrant lignin aromatic structure, the biopolymer compatibility with PBAT has allowed manufacturing plastic composite films containing biodegradable PBAT and starch polymers, and biocompatible BPs, which have the bifunctional properties to protect the soil and stimulate crop production.

## 2. Materials and Methods

### 2.1. Materials and Treatments

The BMP_Z_ BPs were available from previous work [10]. The post-harvest tomato plants (Lycopersicon esculentum Cv. Naomi F1) were hydrolysed 4 h with KOH solution at pH 13, at 60 °C and a 4 (*v*/*w*) alkaline water/solid BMPz ratio. A typical mixture contained 480 L water, 120 g BMPz, and 7 g KOH. The liquid/solid hydrolysate was allowed to settle to separate the supernatant liquid phase from the insoluble matter. The recovered liquid hydrolysate was filtered through a polysulphone membrane with a 5 kDa molecular cut, and the membrane retentate was dried in a ventilated oven at 60 °C. The FORSUD BPs were the dry hydrolysate obtained from the food waste anaerobic digestate produced at Acea Pinerolese waste treatment plant in Pinerolo, Italy. The hydrolysate was obtained under the same experimental conditions as above, except for the 90 °C hydrolysis reaction temperature. PBAT, grade Ecoflex F Blend C1200 from BASF (Ludwigshafen, Germany), was supplied by Société D’Extrusion Du Polyéthylène A. Barbier (Sainte-Sigolène, France). Unless otherwise indicated, all other chemicals were purchased from Thermo Fisher Scientific Inc. (Oxoid Limited, Basingstoke, Hampshire, UK), VWR Chemicals (Radnor, PA, USA), Sigma-Aldrich (St. Louis, MO, USA), and Millipore Corp. (Bedford, MA, USA), and were of analytical grade or higher. Ultrapure water was obtained from a Millipore Corp. Milli-Q water purification system.

### 2.2. Manufacture and Characterisation of Single-Layer Films

#### 2.2.1. Twin-Screw Extrusion Compounding

FORSUD and BMPz BPs were ground using an Electra (Poudenas, France) F4 hammer mill fitted with a 1 mm screen, and then sieved in a RITEC (Saint-Paul-Trois-Chateaux, France) 600 vibrating sieve shaker fitted with a 500 µm screen. They were then dried at 80 °C for one night in a ventilated oven. The BPs were then mixed with PBAT through twin-screw extrusion compounding. Moisture at the moment of compounding (measured according to ISO 665:2000 standard) [21] was negligible for PBAT, 1.21 ± 0.25% for FORSUD BPs, and 2.26 ± 0.03% for BMPz BPs. Compounding was conducted using a Clextral (Firminy, France) Evolum HT 53 twin-screw extruder made of co-rotating and intermeshing screws, and nine consecutive modules (53 mm for the screw diameter (D) and 4 D for each module length, for a total barrel length of 36 D). PBAT was introduced in module 1, and the introduction of the BP filler was made at the level of module 6 thanks to a side feeder. The screw profile was previously optimised, and inspired by that used in a previous study [22]. The temperature profile along the barrel (from module 1 to module 9) was 25-40-125-150-150-140-140-135-135 °C, and the temperature at the die was 135 °C. The die was made of 2 holes, each 3 mm in diameter. Then, the rods were continuously cooled with cold water in a cooling tank before being granulated using a granulator knife. The compounded granules were used to manufacture the single-layer and the three-layer films, according to the respective procedures described below.

#### 2.2.2. Melt Rheology Measurements

Melt rheology measurements were conducted on neat PBAT (before and after extrusion), and on all the compounds at 150 °C using a Thermo Scientific Haake (Karlsruhe, Germany) MiniLab micro-compounder. Seven screw rotation speeds were tested, i.e., 50, 75, 100, 125, 150, 175 and 200 rpm, corresponding to applied shear rates varying from 177 up to 1244 s^−1^, and melt rheology measurements were made once the motor torque has stabilised for each shear rate step. Apparent viscosity was measured every 3 s for 30 s at each shear rate step, and the average value calculated from the 10 measurements was used to plot the melt rheology curve, which shows how apparent viscosity changes with shear rate. Modelling of the melt rheology curves was conducted using the Ostwald and de Waele’s power law (Equation (1)):

(1) $$\eta =K\times {\left|\dot{\gamma }\right|}^{m-1}$$

where η is the apparent viscosity (in Pa s), $\dot{\gamma }$ is the shear rate (in s^−1^), K is the consistency (in Pa s^m^), and m is the power law index (i.e., pseudoplasticity index). The m and K parameters were calculated from the data fittings, and the related correlation coefficient (R^2^) was determined at the same time, as in previous work [22]. For each formulation, melt rhelogy measurements were conducted twice, and results for m, K, and R^2^ are presented as mean values ± standard deviations.

#### 2.2.3. Production and Tensile Test of Single–Layer Sheet Films

Production and tensile tests of single-layer sheet films were carried out at Laboratoire de Chimie Agro-industrielle, Université de Toulouse, INRAE, ENSIACET (Toulouse, France).

Sheet films were produced from neat PBAT and from the different twin-screw extrusion (TSE) compounded granules (see Section 2.2.1) using a Scamex (Isques, France) 30–26 single-screw extruder (SSE) with a 26 length-to-diameter ratio, and a 30 mm screw diameter. The temperature profile was 105 °C in the conveying zone, 120 °C in the melting zone, 130 °C in the kneading zone, and 130 °C at the outlet die. The screw rotation speed was 15 rpm. For the die, the lip height, measured with an electronic digital sliding calliper, was 0.45 ± 0.05 mm, and it was 10 cm in width. After SSE stabilisation, all films were produced over 5 min. Material pressure and temperature at the die were measured during sampling thanks to two sensors, and the values obtained were recorded every 15 s (i.e., 20 measurements per film sampling). Results for material pressure and temperature are presented as mean values ± standard deviations. Calendar speed was adjusted to have a film thickness near 330 µm for all composite formulations (see Table 1 in the Results and Discussion section for further experimental details). The thickness and width of the films were then measured using the same electronic digital sliding calliper, and 25 measurements were made for each film. Results are presented as mean values ± standard deviations.

Tensile tests on films produced by SSE were conducted using the ISO 527-3 standard [23]. Before these tests, films were conditioned in a climatic chamber at 25 °C and 60% relative humidity (RH) for three weeks. They were performed at room temperature with an Instron (Norwood, MA, USA) 4204 universal testing machine fitted with a crosshead maximum strength capacity of 500 N. A fixed crosshead rate of 20 mm/min was utilised in all cases. Specimens used were cut from the previously conditioned extruded films using a craft knife. They were rectangular (20 mm in width, and 150 mm in length). All measurements were conducted on five specimens.

### 2.3. Manufacture and Characterisation of Three-Layer Blown Films

**Figure 1:**
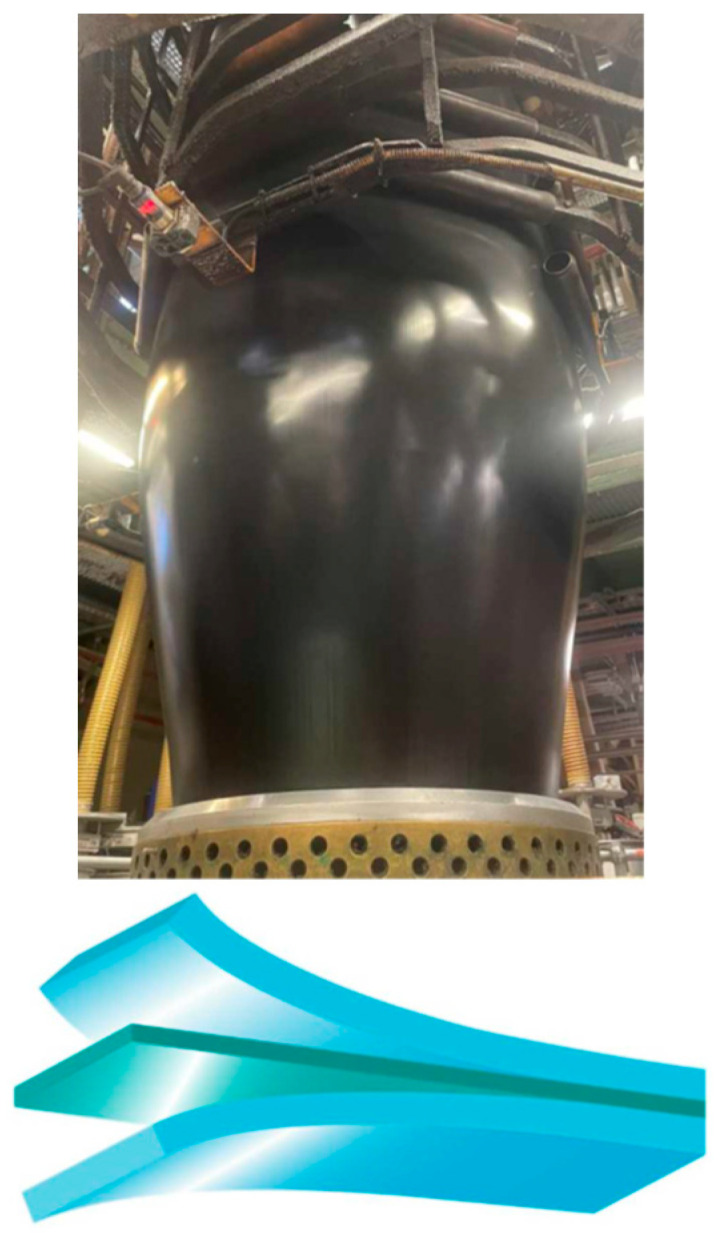
Blown film (**top**) and three-layer configuration (**down**) for Starch-PBAT composites containing PBAT-BPs in the core layer between the two starch skin layers. See also Figure 2 in Section 2.5.

Blown film production was carried out at Société D’Extrusion Du Polyéthylène A. Barbier (Sainte-Sigolène, France), in a Labtech three-layer co-extruder with a 30 length-to-diameter ratio, a 90 mm screw diameter for the core layer, and a 70 mm screw diameter for the skin layers (Figure 1). Looking at these three layers, on the one hand, the core layer was a blend made of 70% (*w*/*w*) neat PBAT, 20% (*w*/*w*) TSE compounded granules (see Section 2.2.1.), and 10% (*w*/*w*) black colourant. On the other hand, both skin layers were mixtures of a starch compound and a black colourant (90% (*w*/*w*) and 10% (*w*/*w*), respectively). Here, the black dye was added to enhance the agronomic properties during in-field application, specifically to make the film highly effective at suppressing weed growth and warming the soil. It is important to note that the black dye is manufactured from materials that are biodegradable in the soil and is even certified as SOIL COMPOST. In these proportions, it has no impact on the film’s mechanical properties. The core layer represented 50% (*w*/*w*) of the three-layer blown film, and the mass proportion of the two skin layers was 25% (*w*/*w*) each. The temperature profile was 105 °C in the conveying zone, 130 °C in the melting zone, 130 °C in the kneading zone, and 130 °C at the outlet die. The die was 30 cm in width. Roll speed was adjusted to have a film thickness near 20 µm for all composite formulations. Tensile tests were conducted using the ISO 527-3 standard once the three-layer blown films had been conditioned in a climatic chamber (25 °C, 60% RH) for three weeks. They were performed at room temperature with a Shimadzu (Kyoto, Japan) testing machine fitted with a crosshead maximum strength capacity of 500 N. A fixed crosshead rate of 500 mm/min as proposed in ISO 527-3 was utilised in all cases. Specimens used were cut from the previously conditioned three-layer blown films using a craft knife. They were rectangular (15 mm in width, and 50 mm in length). All measurements were conducted on five specimens.

### 2.4. In Vitro Cress Seed Germination

Seeds of garden cress (*Lepidium sativum* L.), commonly used as a culinary herb, were obtained from a commercial firm and used as plant material. The mono- and three-layer films were then exposed to water for 1, 4 and 8 days (Table 1). Specifically, T1 corresponded to one day of exposure, which was the time required for seed germination. T4 consisted of four days of exposure prior to seed addition, followed by one day for seed germination. Similarly, T8 consisted of eight days of exposure prior to seed addition, followed by one day for seed germination. The test PBAT single-layer films contained 4, 10, 12, 14 and 16% of FORSUD or BMPz BPs, whereas the test PBAT three-layer films contained 1, 1.5 and 4% FORSUD or only 4% BMPz, referring to the total mass of the sample. The PBAT single-layer and PBAT three-layer containing no BPs were included as control films, respectively, for the single-layer and three-layer films containing the BPs (Table 1). Tests for single-layer films containing FORSUD and BMPz were performed in two separate batches at different dates; therefore, two different controls of PBAT single-layer films were introduced in each experimental batch. These were PBATc1 and PBATc2 for PBAT- FORSUD single-layer and PBAT-BMPz single-layer films, respectively, as detailed in Table 1. The experiments were carried out at the Department of Agriculture, Food and Environment at the University of Catania in October and November 2025.

The germination phase of the study was assessed in Petri dishes by evaluating the effects of the bioplastic films on cress seeds, representing the most sensitive species to many toxic compounds, which have been commonly used in germination testing. The seeds were initially sterilised to eliminate potential microbial contaminants, by immersing them in a 3% sodium hypochlorite solution for five minutes, followed by multiple rinses with distilled water to remove any residual disinfectant [24]. After disinfection, 20 seeds were carefully placed in each Petri dish with one Whatman No. 1 filter paper. To assess the impact of exposure to bioplastic films on seed germination, a layer of each tested film was placed under the filter paper. Each treatment was carried out in triplicate, ensuring statistically reliable results. The Petri dishes were then arranged in a completely randomised design to minimise positional effects. This experimental setup enabled the dose-dependent effects of FORSUD and BMPz treatments on seed germination to be evaluated in a controlled and systematic way, allowing a comprehensive assessment of their phytotoxic or biostimulant impact to be made. To ensure uniform environmental conditions across all treatment groups, all Petri dishes were placed in a controlled climate chamber. The temperature was kept constant at 25 ± 1 °C and the seeds were kept in complete darkness throughout the germination period to prevent light influencing germination responses. The seeds were considered to have germinated when a radicle of at least 2 mm had emerged. The length of the radicle was measured for each seedling one day after starting, using a digital calliper to the nearest 0.01 mm. Based on these parameters, several germination indices, such as the germination percentage (GP), germination index (GI), seedling vigour index (SVI) and relative growth of the radicle (RGR), were calculated according to the following equations:

(2) $$GP\left(\%\right)=\frac{number\;of\;germinated\;seeds}{number\;of\;tested\;seeds}\times 100$$

(3) $$GI\left(\%\right)=\frac{germinated\;seeds\left(sample\right)\times radicle\;lenght\left(sample\right)}{mean\;germinated\;seeds\left(negative\;control\right)\times mean\;radicle\;lenght\left(negative\;control\right)}\times 100$$

(4) $$\mathrm{SVI}=GP\left(sample\right)\times radicle\;lenght\left(sample\right)$$

(5) $$RGR\left(\%\right)=\frac{radicle\;lenght\left(sample\right)}{mean\;radicle\;lenght\left(negative\;control\right)}\times 100$$

The weight loss of the films was also evaluated during the germination experiment for all triplicates. Each film sample was weighed before the start of the exposure to water (initial weight) and again at the end of the experimental period (final weight), after gently removing surface moisture. Measurements were performed using an analytical balance with a precision of 0.001 g. The weight loss was expressed as the percentage reduction relative to the initial weight. All experiments were performed in triplicate and the results, expressed as the mean ± standard error of the mean, were analysed using a one-way analysis of variance (ANOVA), followed by Tukey’s honest significance difference (HSD) post hoc test with a 95% confidence interval, in IBM SPSS Statistics 22.

### 2.5. FORSUD BP Three-Layer Film Deterioration During In-Field Cultivation of Horticulture Plants

**Figure 2:**
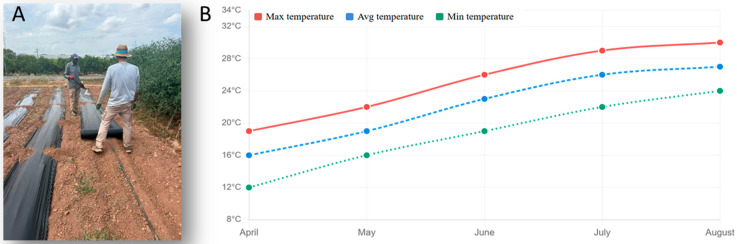
Starch-PBAT-FORSUD BP three-layer film on-field installation (**A**) and average, minimum and maximum temperatures in Almeria (Spain) during experimental period (**B**).

Film degradation was evaluated according to known methods, i.e., by visual analysis and mass loss [25]. Figure 2A shows the mulch film on-field installation. The degradation of the Starch-PBAT-FORSUD BP three-layer films was monitored over 165 days of field exposure throughout the cropping season of four different crops, eggplants, tomato, green pepper and cucumber. The inclusion of the four plants in the experimental plan was expected to allow assessing whether the replicability of the film deterioration was affected by the plant species. Crops were grown on different soil lots covered with (i) conventional polyethylene film (Conventional PE) and with Starch-PBAT-FORSUD BP three-layer films containing (ii) 1% (Starch-PBAT-1% FORSUD BPs) and (iii) 1.5% BPs (Starch-PBAT-1.5% FORSUD BPs). Crops were cultivated according to a previously reported procedure [26]. In particular, plant cultivation was carried out with a randomised complete block (RCB) experimental design, with a plant rate of 25,000 plants/ha, and a row spacing of 0.4 m + 1.2 m, in accordance with agricultural practices widely adopted by local farmers in the Mediterranean area. Crops were regularly irrigated during the cultivation period and climatic data such as maximum temperature, average temperature and minimum temperature were also recorded (Figure 2B). The substrate was a sandy soil (86.2% sand, 7.1% silt and 6.7% clay) characterised by the following parameters: pH 7.85, electrical conductivity 1513 mS/cm, organic carbon 0.87 *w*/*w* %, cation exchange capacity 8.59 cmol/kg, total nitrogen 20.42 mg/kg, phosphorus 5.43 mg/kg, and potassium 8.67 mg/kg. The experiment design included a RCB pattern comprising 12 whole plots of around 1000 m^2^ each, covered with the above three films, and tested for the cultivation of the above four crops. Visual assessments of deterioration were performed periodically on each plot according to a previously published procedure [27]. Specifically, within a designated 1.5 m × 0.6 m bed area in the centre of each mulching film strip, the total number of rips, tears, and holes (RTH) was counted and percent visually observed deterioration (PVD) was calculated, with values ranging from 0% for completely intact films to 100% for fully deteriorated films. Visual assessments of deterioration were performed for each film installed during cultivation (Figure 2A), and the results are expressed as the average percentage degradation calculated from all recorded values. Total yield was finally determined at the end of the cropping period, calculating the weight of total fruits collected under each mulching film, and expressed as fruit weight (kg/ha).

## 3. Results and Discussion

### 3.1. Film Production Data

Table 2 reports the experimental details of the film production through single-screw extrusion. For all films, their production was stable over time, as evidenced by the low standard deviation for the material pressure at the die during sampling. The following observations were made during the film production through SSE, and these are in accordance with the melt rheology data shown in Figure 2 and Table 3 below. Firstly, self-heating was observed at the die, especially for neat PBAT, which is more viscous. Secondly, for both BP fillers, material pressure at the die decreased in comparison with that for neat PBAT, in accordance with the fluidizing effect of the BP fillers on PBAT. Lastly, and for the same reason, the higher the filler incorporation rate, the lower the material pressure at the die, as melt rheology decreases with an increased incorporation rate for both fillers.

### 3.2. Melt Rheology and Mechanical Properties of PBAT-BP Single-Layer Films

The melt rheology, and mechanical data consistently provide information on the compatibility of BPs with the PBAT matrix.

**Figure 3:**
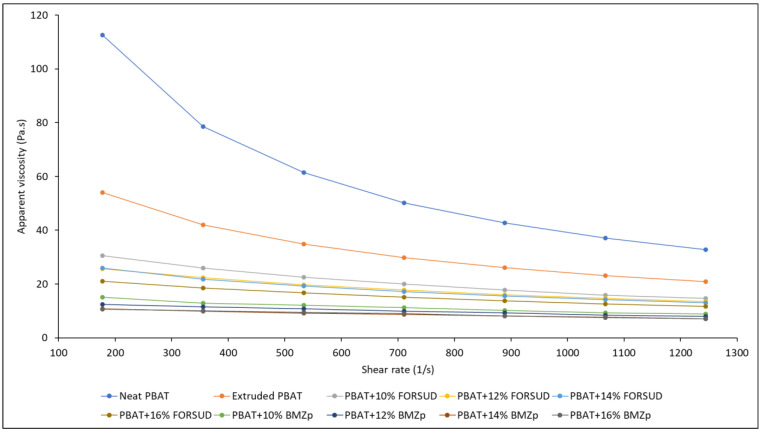
Apparent viscosity as a function of shear rate for neat PBAT (before and after extrusion), and all formulations.

Figure 3 shows that PBAT (neat or extruded) and all compounds exhibit a shear thinning behaviour, i.e., the higher the shear rate, the lower the apparent viscosity. Extruded PBAT has a lower viscosity than the neat one, which could be synonymous with a slight degradation of the polymer matrix during passage through the twin-screw extruder. Both BP fillers have a fluidizing effect on molten PBAT, and the higher the filler incorporation rate, the lower the viscosity. In addition, with the same filler incorporation rate, the compound containing BMPz has a lower viscosity than that containing FORSUD, meaning that, of the two fillers, BMPz is the one with the strongest fluidizing effect on PBAT.

Table 3 shows that the trend of the K and m parameters originating from the power law of Ostwald and de Waele (Equation (1)) is in perfect accordance with that of the apparent viscosity (Figure 3), i.e., the lower the viscosity, the lower the consistency (K) and the higher the pseudo-plasticity index (m). A similar pattern to that shown in Figure 3 and Table 3, respectively, for the apparent viscosity and for the trend of K and m parameters as a function of the shear rate and the content of the BP filler, was reported in a previous study for EVOH-FORSUD BP blends [9]. Yet, the PBAT and EVOH blends at the same FORSUD BP content show significant quantitative difference. First, the apparent viscosity at 177 s^−1^ shear rate for neat EVOH was measured about 96 Pa.s at 200 °C instead of only 54 Pa.s for extruded PBAT (Figure 3), despite the much lower 150 °C temperature at which the PBAT viscosity was measured (see Section 2.2.2). Then, the apparent viscosity of the EVOH-FORSUD BP blends was found to decrease to 80 Pa.s for the blends containing 5–15% FORSUD BPs, and then to increase to about 96 Pa.s at 16% FORSUD BP content. By comparison, the apparent viscosity for the PBAT-FORSUD BP blends (Figure 2), upon increasing the filler content from 10 to 16%, decreases continuously from 31 to 21 Pa.s. The higher relative decrease in the apparent viscosity of the PBAT blends, i.e., 61% of the value for the neat polymer matrix, compared to only 17% reduction for EVOH containing 5–15% FORSUD BPs, is likely related to the stronger interaction of the FORSUD filler with PBAT than with EVOH.

A detailed discussion of the reasons for viscosity to decrease as a function of the blends nature and composition is given in the paper by Franzoso et al [9]. The FORSUD [9] and BMPz [10] BPs, mixes of molecules with molecular weights ranging from 5 to over 750 kDa, constituted by aliphatic and aromatic carbon types substituted by acid and basic functional groups, may react and/or interact with the PBAT and EVOH matrices in a number of ways, e.g., by forming a new copolymer, or by dissolving in the synthetic polymer matrix by electrostatic and/or H-bonding interactions. Depending on the type of interaction, the BP filler molecules may occupy the space between polymer chains, increasing their intermolecular distance and therefore permitting higher mobility. In this fashion, the matrix macromolecules may slide within the blend system during the application of shear forces, causing a flow favouring orientation, which subsequently lowers the viscosity of the PBAT and EVOH matrices. The increase in the blend viscosity as observed for the EVOH-BP blends, at BP content > 15%, may be explained with the saturation of the EVOH OH functional groups engaged in H-bonding interaction with the added BP molecules. Under these circumstances, the excess BP molecules tend to aggregate with each other through intramolecular bonds, which causes a reduction in molecular mobility and therefore the increase in viscosity. The higher relative decrease in the apparent viscosity of the PBAT blends, compared to that for the EVOH ones, suggests that more and stronger interactions occur between the functionalised aliphatic and aromatic moieties present in both BP fillers and the PBAT polymer matrix. In this case, there is less saturation of intermolecular contacts between BPs and PBAT, and less formation of BP aggregates occurs. This situation allows a higher number of contacts between BPs and PBAT, which results in the stronger plasticising effect and higher compatibility of the BPs for the PBAT polymer matrix than for the EVOH one. For future work, as the viscosity decrease observed for PBAT inside the BP-based blends could also be the result of a slight hydrolytic and/or thermomechanical degradation of the polymer matrix during the process, it would be interesting to demonstrate if such degradation occurs or not, e.g., by using Size Exclusion Chromatography (SEC) in order to observe any changes in the molecular weight of PBAT following extrusion.

### 3.3. Film Mechanical Properties

Table 4 reports tensile data for the single-layer PBAT films containing 10–16% FORSUD or BMPz BPs, and for the three-layer PBAT films containing (or not containing) FORSUD BPs. Looking at the data in this table, as no compatibilizer was added at the interface between PBAT and the BP filler at the moment of compounding through twin-screw extrusion, an important decrease is observed for the maximum tensile stress of the single-layer films fabricated by SSE from the compounded granules. In accordance with the rheological data trends (Figure 2 and Table 3), it may be observed that the higher the filler incorporation rate, the lower the maximum tensile stress—but BMPz seems, however, to have more compatibility (or, rather, less incompatibility) with PBAT as the decrease in maximum tensile stress is less significant. A decrease in the elongation at break is observed at the same time, and the tensile modulus tends to be more important with BMPz, meaning that the corresponding films are more rigid. In contrast, in the presence of the FORSUD BP filler, the Young’s modulus is highly decreasing, around −75%, relative to the value for neat PBAT, which means that PBAT is becoming much more flexible when filled with FORSUD, regardless of the incorporation rate.

Further support for the above trends was obtained from the mechanical data obtained for the three-layer PBAT film (i.e., the PBAT-0% FORSUD BP film, with no BPs added), and for the three-layer PBAT films containing 1.0 and 1.5% FORSUD BPs. The following effects were observed. Firstly, when comparing neat PBAT (Table 4) with the three-layer Starch-PBAT-0% FORSUD BP film, a decrease in both maximal tensile stress (from 35 MPa to 25.1 MPa) and elongation at break (from 710% to 520%) was observed. This likely means that the interface between the internal PBAT layer and the two external starch ones fragilize the mechanical behaviour of PBAT. These two interfaces are, in fact, of preferred rupture during tensile testing. Secondly, when comparing the 0, 1.0, and 1.5% FORSUD BP three-layer films against the others in Table 4, the tensile strength values of the former ones were found to be much higher, i.e., 23.9–25.1 MPa instead of only 3.8–6.8 MPa for the 10–16% BP films. This result supports the fact that fragility is the consequence of there being no coupling agent at the interface between PBAT and BPs, and therefore the higher the BP content, the more fragile the films.

The authors are aware that comparing the results obtained with the above single-layer and three-layer films is not easy for several reasons. Firstly, moulding processes are not the same, i.e., single-screw extrusion plus calendering for the single-layer films, and blow extrusion for the three-layer ones. The thicknesses are also not the same. Lastly, in the three-layer films, the two external starch films may contribute in different ways to the mechanical strength of the films, depending on the type and content of the BP fillers. A much easier and more informative way is to compare single-layer PBAT-BP films with single-layer EVOH-BP ones [5,8,9]. In this case, the fabrication and BP content of the films are the same, except for the synthetic polymer matrices.

### 3.4. Comparing Single-Layer PBAT-BP Films with Single-Layer EVOH-BP Films (Matrix Effects on Mechanical Properties)

The work reported here is a step of a long-term R&D project started in 2004, with the primary objective to realise a biowaste-based refinery producing value-added chemical specialities, to use in place of the commercial products derived from fossil feedstock. In the specific sector of composite plastic blends, the assessment of the matrix effects on the mechanical properties of the BP blends is of high importance to open new R&D routes for developing and optimising marketable bioplastic materials, which could compete for both cost and performance with current commercial plastics. To this end, the collected data on the single-layer PBAT-BP films in the present work, and the previously published data on the single-layer EVOH-BP films [5,8,9,10], offer a sound opportunity to assess the effects of the two different polymer matrices on the film’s mechanical properties.

The mechanical data for the PBAT (Table 4) and EVOH [5] composites filled with 10% FORSUD or BMPz BPs show, in both cases, quite similar behaviour. The limited compatibility between the matrices and the fillers is responsible for decreases in maximal tensile stress, from 35 MPa to around 6.5 MPa for the PBAT composites (i.e., −80%), and from 55 MPa to around 15 MPa for the EVOH composites (i.e., −70%). The same is true for elongation at break, even if the reduction is much less significant with PBAT, especially in the case of PBAT-FORSUD BPs: 65% reduction in elongation at break for the two EVOH composites (i.e., from 13.75% to 4.75%), 45% reduction for the PBAT composite filled with 10% BMPz (i.e., from 710% to 395%; see Table 4), and only 4% reduction for the PBAT composite filled with 10% FORSUD (i.e., from 710% to 681%; see Table 4). This shows that the PBAT and PBAT-based composites are the best choices for flexible applications like mulch films ones. And, when a filler is added, even if both matrices become less rigid (i.e., decrease in Young’s moduli), their mechanical strength and elongation at break are reduced due to incompatibility between the filler and the matrix. Nevertheless, elongation at break, as shown by the PBAT-BP composite mulch films, and particularly by the PBAT-FORSUD BP ones (i.e., in the 400–680% range), is quite adequate for mulch field functions [28]. The data offer worthwhile R&D scope for improvements of the mechanical data, for example, by compatibilization of the interface thanks to the addition of coupling agents [29].

### 3.5. Testing PBAT-BP Mulch Films in Agriculture

**Figure 4:**
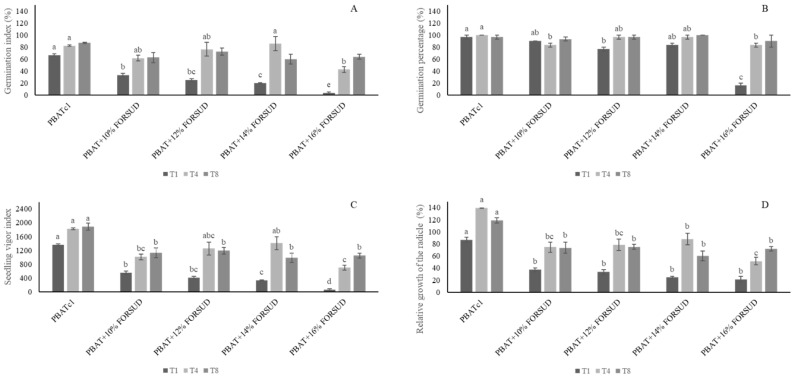
Post hoc effects of single-layer PBAT-FORSUD films on germination index (**A**), germination percentage (**B**), seedling vigour index (**C**) and relative radicle growth (**D**) of cress seeds. Data from two trials ± SEM (standard error of the mean). Means for each parameter were obtained from 3 replicates. According to Tukey’s least significance differences test, values followed by different letters, compared at each time of exposure, are significantly different (*p* < 0.05). Legends as in Table 1. PBATc1 indicates the single-layer PBAT-FORSUD film containing no FORSUD.

**Figure 5:**
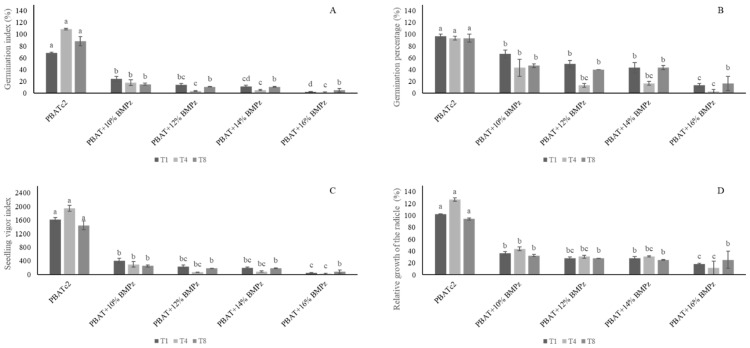
Post hoc effects of single-layer PBAT-BMPz films on germination index (**A**), germination percentage (**B**), seedling vigour index (**C**) and relative radicle growth (**D**) of cress seeds. Data from two trials ± SEM (standard error of the mean). Means for each parameter were obtained from 3 replicates. According to Tukey’s least significance differences test, values followed by different letters, compared at each time of exposure, are significantly different (*p* < 0.05). Legends as in Table 1. PBATc2 indicates the single-layer PBAT-BMPz film containing no BMPz.

**Figure 6:**
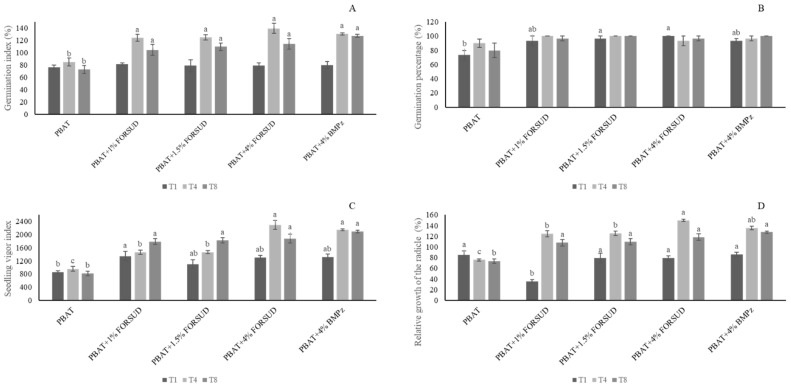
Post hoc effects of three-layer Starch-PBAT-FORSUD films on germination index (**A**), germination percentage (**B**), seedling vigour index (**C**) and relative radicle growth (**D**) of cress seeds. Data from two trials ± SEM (standard error of the mean). Means for each parameter were obtained from 3 replicates. According to Tukey’s least significance differences test, values followed by different letters, compared at each time of exposure, are significantly different (*p* < 0.05). The absence of letters above the bars indicates no statistically significant differences among treatments (*p* ≤ 0.05). Legends as in Table 1. PBAT indicates the three-layer Starch-PBAT-FORSUD film containing no FORSUD.

The effects of the tested films on cress seed germination are shown in Figure 4 (single-layer PBAT-FORSUD films), Figure 5 (single-layer PBAT-BMPz films) and Figure 6 (three-layer Starch-PBAT-FORSUD films). To allow comparisons among films with similar structural compositions, specific reference controls were used, as detailed in the material and methods section.

For single-layer PBAT-FORSUD films (Figure 4), significant differences in the cress seed germination GI parameter were observed among treatments (*p* < 0.05) only at T1 and T4. In detail, increasing FORSUD concentration (10–16%) resulted in a progressive reduction in GI compared with the single-layer PBATc1 control at T1, whereas at T4, only PBAT+16%FORSUD determined a significant reduction in GI compared to the control. The GI decrease indicates an inhibitory effect for the cress seed germination by the FORSUD BPs, which is more evident for the films with the highest 16% FORSUD BP concentration. The GP parameter was generally less affected than GI. In particular, at T1, only the highest FORSUD BP concentration (16%) significantly reduced GP compared with PBATc1. For the SVI parameter, the trend showed that high concentrations of FORSUD (10–16%) led to a significant reduction in SVI at all exposure times with respect to the control, except for PBAT + FORSUD 12 and 14% at T4, which recorded values similar to the control. Finally, the RGR parameter decreased significantly with respect to the control at all FORSUD BP concentrations (10–16%) across all exposure times (T1, T4, and T8).

Similar concentration-dependent trends were observed for the single-layer PBAT-BMPz films (Figure 5). However, the inhibitory effect of BMPz appeared more consistent over time. GI, SVI, and RGR decreased significantly at all concentrations (10–16%) across all exposure times (T1, T4, and T8), compared with the PBATc2 control. As seen with the PBAT-FORSUD films, GP remained the least affected parameter, with significant reductions primarily confined to the highest concentrations.

In contrast to the single-layer films, the three-layer films (Figure 6), which incorporated lower additive concentrations (1–4%), exhibited a markedly different trend. At T1, these films did not greatly differ from the Starch-PBAT three-layer control in terms of GI, SVI, or RGR. Notably, after longer time of pre-exposure to water (T4 and T8), a stimulatory effect on cress seed germination by the Starch-PBAT-BP films emerged. Specifically, the Starch-PBAT-4% FORSUD and Starch-PBAT-4% BMPz films showed significantly higher GI and SVI values compared to the Starch-PBAT three-layer control. This indicates that while high BP concentrations in single-layer films are inhibitory, low BP concentrations in the three-layer film structure can promote seedling development under prolonged exposure conditions.

#### 3.5.1. Characterisation of Films Recovered from Cress Seed Germination Tests

The weight loss of the films recovered from the cress seed germination tests was measured to attempt to understand the reason for their effects on the measured GI, GP, SVI and RGR parameters.

**Figure 7:**
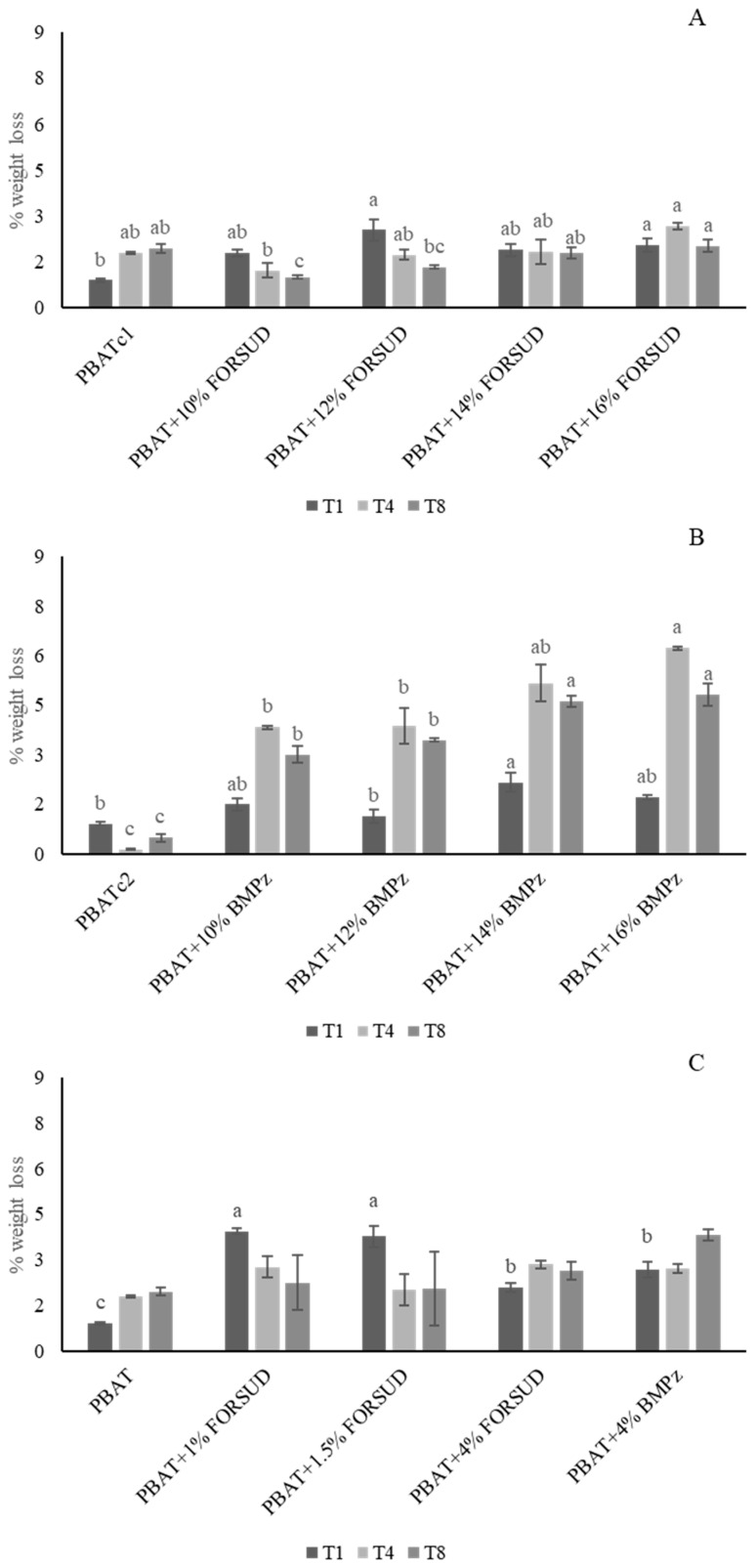
Weight loss during the cress seed germination process for single-layer PBAT-FORSUD BP films (**A**), single-layer PBAT-BMPz BP films (**B**) and three-layer Starch-PBAT-BP films (**C**). Data from two trials ± SEM (standard error of the mean). Means for each parameter were obtained from 3 replicates. According to Tukey’s least significance differences test, values followed by different letters, compared at each time of exposure, are significantly different (*p* < 0.05). The absence of letters above the columns shows the lack of significant differences. Legends as in Table 1 and Figure 4, Figure 5 and Figure 6. PBATc1 and PBATc2 indicate single-layer PBAT-FORSUD BP and PBAT-BMPz BP films containing no BPs, respectively. PBAT indicates three-layer Starch-PBAT-BP film containing no BPs.

**Figure 8:**
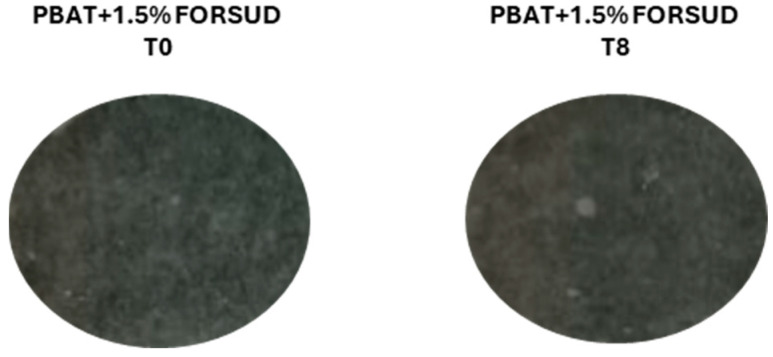
Aspect of PBAT+ 1.5% FORSUD three-layer films at the beginning (T0) and after 8 days’ immersion in the seed germination aqueous medium.

Figure 7 reports the results obtained for the single-layer and three-layer films containing FORSUD and BMPz BPs recovered from the tests at T1, T4, and T8 exposure time. It may be observed that the single-layer films containing the BMPz BPs (Figure 7B) exhibit a more pronounced and time-dependent increase in weight loss, particularly at 14–16% concentrations at T4 and T8, compared to the single-layer films containing the FORSUD BPs. In both cases, the controls for monolayer films (PBATc1 in Figure 7A and PBATc2 in Figure 7B) exhibited the lowest weight loss. For the three-layer films (Figure 7C), although marked differences were observed at T1, the differences at T4 and T8 were more limited. Overall, the highest weight losses of the single-layer films during the in vitro cress germination trials were about 6%, and the aspect of the recovered films did not differ from the starting films, as shown, for example, in Figure 8.

#### 3.5.2. Deterioration/Biodegradation of Three-Layer Starch-PBAT-FORSUD Films During On-Field Cultivation of Horticulture Plants and Effects on Crop Production

**Figure 9:**
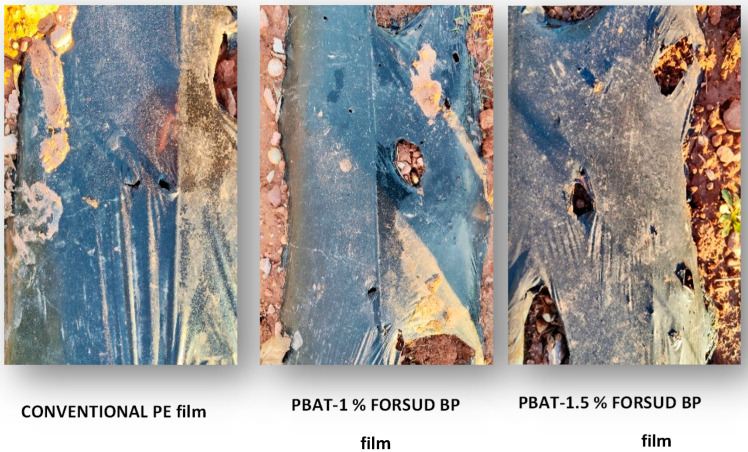
Aspect of conventional polyethylene (PE) and PBAT-FORSUD three-layer films after 165 days on-field operation.

**Figure 10:**
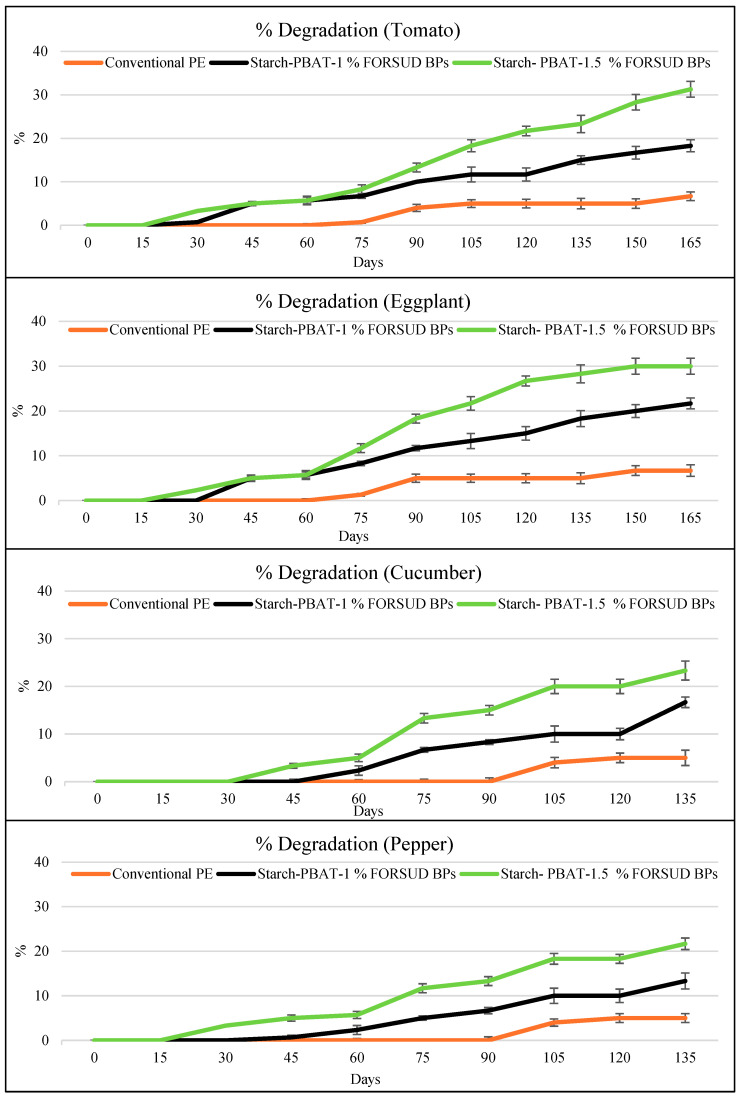
Degradation % of conventional polyethylene (PE) film, and PBAT-1 and -1.5% FORSUD three-layer films as a function of time (days) and the cultivated plants (tomato, eggplant, cucumber and pepper). Data from three visualisations ± SEM (standard error of the mean).

**Figure 11:**
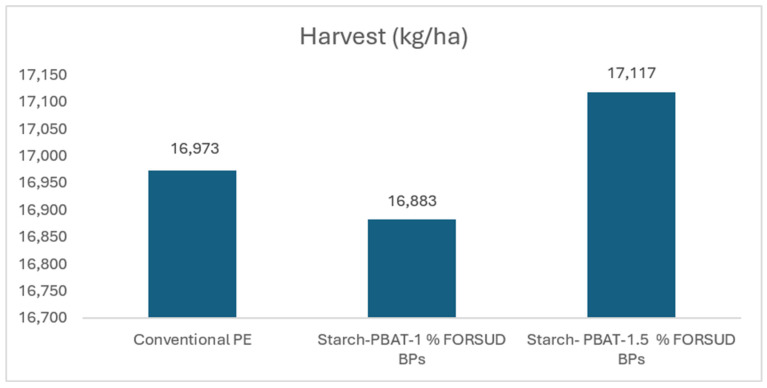
Production of tomato cultivated on soil covered with conventional polyethylene (PE) mulch film, and three-layer Starch-PBAT films containing 1 and 1.5% FORSUD three-layer films.

Figure 9, Figure 10 and Figure 11 show the results of the on-field biodegradation for the three-layer Starch-PBAT films containing 1 and 1.5% FORSUD BPs compared to a conventional polyethylene (PE) mulch film.

Figure 9, compared to Figure 2, shows how after 165 days from their installation, the mulch films appear consistently deteriorated. Also, the photo of the three film samples in Figure 9 shows that, as expected [3], the three-layer Starch-PBAT-FORSUD BP mulch films appear consistently more damaged than the PE mulch film, with the Starch-PBAT-1.5% FORSUD BPs looking the most damaged. This qualitative difference appears substantiated from the quantitative data reported in Figure 10, which were obtained counting the number of rips, tears, and holes in the samples taken periodically in the different lots cultivated with the four different horticulture plants on soil covered with the above PE and Starch-PBAT-FORSUD BP mulch films, according to the method [27] reported in Section 2.

The applied method [27] to obtain the data reported in Figure 10 does not allow assessing whether the observed changes in the aspect of the tested films are due to the chemical and/or physical deterioration of the mulch films caused by atmospheric factors or to the biodegradation caused by soil micro-organisms. Although Starch-PBAT three-layer mulch films are expected to be more biodegradable than the neat PBAT polymer [3], the mechanical data in Table 4 demonstrate that the two external starch layers (Figure 1) in the three-layer film weaken the mechanical behaviour of the pristine neat PBAT polymer. In any case, from the practical point of view of the end-user farmer, biodegradable mulches must maintain their functionality of protecting the soil during the plant growing season to improve the plant performance. The results reported in Figure 10 show that the degradation level of the PE film after 165 days ranged from 5 to 6.7%. By comparison, for the Starch-PBAT-1% FORSUD three-layer film, the degradation level ranged from 13 to 22%, and for the Starch-PBAT-1.5% FORSUD three-layer film, the degradation level ranged from 22 to 32%. Also, it may be observed that the kinetics of the degradation increases significantly in the order PE < Starch-PBAT-1% FORSUD < Starch-PBAT-1.5% FORSUD. The observed degradation differences among the mulch films do not appear to significantly affect the crop production, as shown in Figure 11 reporting the example of tomato crop production, for which the 1.5% FORSUD three-layer film shows an apparent increase in tomato production. This result appears consistent with previous work, which has assessed the bio-stimulant effect of BPs for a high number of plant species [1,26], along with a parallel effect on the reduction in mineral fertilisers and their environmental impact [30]. Unfortunately, yield data were collected only for tomato, as eggplant, cucumber, and pepper crops were affected by pathogen outbreaks towards the end of the growing season, which would have affected the yield measurements. Since tomato yield data already provide a representative indication of the overall crop performance trend, and given that yield assessment was not a primary objective of this study, yield data for the remaining crops were therefore not reported.

#### 3.5.3. Understanding PBAT-BP Mulch Film Effects and Behaviour in Agriculture

Germination assays revealed a concentration-dependent response of *Lepidium sativum* L. to the tested films. In single-layer films, higher concentrations of both FORSUD and BMPz (10–16%) significantly reduced the germination index (GI), seedling vigour index (SVI) and relative radicle growth (RGR), while the germination percentage (GP) remained largely unaffected. This pattern suggests that the inhibitory effects primarily affected post-germinative development, particularly radicle elongation, rather than the germination process itself. Similar responses have been reported for other bio-based materials, where high filler content promotes the release of soluble organic fractions that can alter osmotic conditions and negatively impact early seedling growth. Conversely, lower FORSUD or BMPz concentrations (1–4%) incorporated into three-layer films did not negatively affect germination parameters, and in some cases resulted in significantly higher GI values after longer exposure times (T4 and T8) compared with the control film. This behaviour is consistent with previous studies reporting that bio-products derived from municipal bio-waste can exert dose-dependent biological effects, promoting seed germination at moderate concentrations, while becoming inhibitory at higher levels. The improved performance observed in the three-layer mulch film configuration after prolonged hydration further suggests that the biological response may depend on the release kinetics of soluble compounds from the polymer matrix. In the three-layer film structures, the external starch layers may regulate to some extent the release of BPs to the soil, preventing crop exposure to initially high BP concentrations and maintaining levels that are compatible with early plant development.

The above results appear to point out that BP solubility and release kinetics in the cultivation substrate is a key issue for the performance of composite materials used in agriculture as controlled-release fertilisers (CRFs). Composite FORSUD BP compounds had previously been manufactured by a continuous twin-screw extrusion process similar to the one described in Section 2.2.1. These products, in pellet form, contained 90% sunflower protein concentrate as matrix polymer together with 5–10% FORSUD BPs and/or urea. They were demonstrated as efficient eco-friendly controlled-release fertilisers (CRFs) for the cultivation of spinach. In this case study, the BPs allowed controlling the formation of ammonia from urea hydrolysis and the release of organic nitrogen, yielding the safest crop coupled with high biomass production. In the case of the present work, both the single-layer and three-layer films were manufactured using FORSUD BP compounds containing PBAT as a matrix polymer. Analysis of the data in Figure 7, Figure 8, Figure 9 and Figure 10 supports the release of BPs from the PBAT polymer matrix. Comparing Figure 7 and Figure 8 with Figure 9 and Figure 10, it can be reasonably inferred that the aspect (Figure 8) and weight losses (Figure 7) of the PBAT-BP mono-layer mulch films, at 1–8 days’ contact with the cress seed germination medium, are more likely consistent with the release of BPs to the cress germination medium. On the contrary, the aspect (Figure 9) and high degradation level (Figure 10) of the Starch-PBAT-FORSUD BP three-layer films during 165 days of on-field operation can only be justified with the high mass loss of the organic PBAT matrix hosting the small amount of BP. The data in Figure 10 also seem to point out that the type of plant species may affect the film deterioration level. This effect seems much higher for the Starch-PBAT-FORSUD three-layer films compared to the conventional PE films. The collected data do not allow a definite assessment and explanation of this effect, but they offer interesting scope for further work in this direction.

## 4. Concluding Remarks

The most important information in the present work is the disclosure of the new PBAT-BP mulch film exhibiting the dual functions [31] of protecting the soil and stimulating crop production. In the composite film, PBAT contributes the basic plastic, mechanical and biodegradability properties, which guarantee the adequate eco-friendly conventional mulch field functions over the lifespan of the plant growth and crop production cycle, and the BP filler performs as slow-release active principles affecting the performance of the cultivated plants. This exploitable finding offers worthwhile scope for further R&D work, particularly in the outlook of realising a bio-waste-based refinery, insofar as it allows widening the range of renewable chemical specialities potentially producible by the envisioned biowaste-based refinery. Additional studies are necessary, however, in order to elucidate and address a number of critical issues disclosed in the present work. The first issue regards the poor plastic properties of the BPs caused by the presence of rigid recalcitrant lignin-like C moieties in the BP chemical composition. This drawback may potentially be overcome by oxidation of the BPs [1] to convert the rigid lignin-like structure containing condensed aromatic rings to a softer aliphatic-aromatic structure, as exemplified in the PBAT polymer. The oxidised BPs (OBPs) might allow producing new plastic materials, where the OBPs could perform as a polymer matrix and contribute the necessary basic plastic, mechanical and biodegradability properties, and in the specific case of mulch films, also the bio-stimulant function for plant growth and crop production.

A second important piece of information in the present work is that the BP fillers in the composite mulch films were sourced from urban and agriculture bio-waste, which proposes the potential of these bio-wastes as feedstock for the envisioned bio-refinery. Compared to the FORSUD BPs, the BMPz BPs seem to be more compatible with the PBAT matrix. However, based on the limitations of the collected data, it is difficult to assess which films have the best potential properties and performance. Further work is necessary to integrate the reported results. For example, Scanning Electron Microscopy (SEM) conducted on films could confirm that the BP particles are well dispersed in PBAT. In parallel, SEC measurements would help to ensure that no reduction in the molecular weight of PBAT is observed after extrusion, which could indicate degradation of the PBAT polymer matrix during the processing stage. Additional characterizations on the three-layer blown films, such as tear resistance, puncture resistance, UV ageing, biodegradation in soil, or assessment of residues after the growing season, would also be worth performing, as they are of particular importance for the practical application of mulch films. This is also true in relation to the after-film exposure in the seed germination and in-field horticulture test, where in principle chemical analyses might confirm that the observed effects resulted from the release of BPs. In practice, the identification of BPs within the test medium is very difficult due to the very complex heterogeneous chemical composition of BPs, which requires a costly protocol involving several different instrumental and wet chemical analyses [1], and is likely to produce doubtful results. In addition to the release of BPs, another issue to be addressed by further research is to clearly distinguish the mechanical degradation of the films from microbiological biodegradation. This would add further complexity to the analytical protocol, as it would require the specific development of a dedicated microbiological analysis to assess the possible effects of the BPs’ interaction with, and effects on, the soil local microbial community as observed in other cases [32]. Nevertheless, due to the importance of BPs as value-added multifunctional bio-products, the development of an improved analytical protocol to identify the BPs during and after their service life, and their chemical and/or biochemical effects, could offer scope for specific dedicated projects.

It appears, therefore, that after all, the present work offers interesting and worthwhile scope for short- and long-term R&D addressing the above process and product criticalities. Specifically, in the short term, the compatibility of the PBAT polymer matrix and BP filler PBAT-BP mulch films could be improved through the use of coupling agents [29,33,34] and a more comprehensive characterisation of the materials during practical applications. In the long term, the BP oxidation process could be developed to manufacture new plastic materials containing OBPs as a polymer matrix and slow-release agent, which in the case of mulch materials, could stimulate plant performance.

## Figures and Tables

**Table 1:** Experimental set-up and legend of film treatments.

Films	Concentration BPs *w*/*w* %	Label Treatment	Time of Exposure of Films to the Water
Control film PBAT-FORSUD single-layer	0	PBATc1 ^a^	T1—1 day T4—4 days T8—8 days
Test films PBAT-FORSUD single-layer	10	PBAT+10% FORSUD	T1—1 day T4—4 days T8—8 days
	12	PBAT+12% FORSUD	
	14	PBAT+14% FORSUD	
	16	PBAT+16% FORSUD	
Control film PBAT-BMPz single-layer	0	PBATc2 ^a^	T1—1 day T4—4 days T8—8 days
Test Films PBAT-BMPz single-layer	10	PBAT+10% BMPz	T1—1 day T4—4 days T8—8 days
	12	PBAT+12% BMPz	
	14	PBAT+14% BMPz	
	16	PBAT+16% BMPz	
Control film PBAT three-layer	0	PBAT	T1—1 day T4—4 days T8—8 days
Test films PBAT-FORSUD three-layer	1	PBAT+1% FORSUD	T1—1 day T4—4 days T8—8 days
	1.5	PBAT+1.5% FORSUD	
	4	PBAT+4% FORSUD	
Test film PBAT-BMPz three-layer	4	PBAT+4% BMPz	T1—1 day T4—4 days T8—8 days

^a^ PBAT-FORSUD single-layer and PBAT-BMPz single-layer tests were performed in two separate batches at different dates. Therefore, two different samples of PBAT single-layer films were introduced in each experimental batch to perform as control films, one for the PBAT-FORSUD single-layer trials and the other for the PBAT-BMPz single-layer trials. These two control samples are indicated in the following Section 3 Figures as PBATc1 single-layer and PBATc2 single-layer.

**Table 2:** Data related to the production of the single-layer PBAT-BP mulch films using SSE.

Formulation	Calendar Speed (m/min)	Material Pressure at the Die (bars) ^a^	Material Temperature at the Die (°C) ^a^	Thickness (µm) ^b^	Width (µm) ^b^
Neat PBAT	5	35 ± 1	192 ± 2	243 ± 7	28 ± 1
PBAT + 10% FORSUD	6	28 ± 2	141 ± 2	330 ± 10	25 ± 1
PBAT + 12% FORSUD	8	25 ± 2	141 ± 2	336 ± 11	25 ± 1
PBAT + 14% FORSUD	10	21 ± 2	141 ± 2	336 ± 18	21 ± 2
PBAT + 16% FORSUD	10	18 ± 2	140 ± 1	340 ± 15	21 ± 1
PBAT + 10% BMPz	6	31 ± 2	137 ± 2	337 ± 15	34 ± 1
PBAT + 12% BMPz	6	28 ± 1	136 ± 2	347 ± 8	37 ± 1
PBAT + 14% BMPz	8	22 ± 2	135 ± 2	321 ± 14	32 ± 1
PBAT + 16% BMPz	7	20 ± 1	134 ± 1	335 ± 13	33 ± 1

^a^ Mean values and standard deviations for material pressure and temperature at the die originate from 20 measurements. Percent values refer to the total mass of the sample. ^b^ Mean values and standard deviations for film thickness and width originate from 25 measurements.

**Table 3:** Consistency (K), pseudoplasticity index (m), and related correlation coefficient (R^2^) reflecting the rheological behaviour in the molten phase of neat PBAT (before and after extrusion), and all formulations according to the Ostwald and de Waele’s power law model.

Formulation	K (Pa.s^m^) ^a^	m ^a^	R^2 a^
PBAT (before extrusion)	3189 ± 28	0.36 ± 0.00	0.9927 ± 0.0002
PBAT (after extrusion)	719 ± 9	0.51 ± 0.00	0.9856 ± 0.0010
PBAT + 10% FORSUD	232 ± 1	0.62 ± 0.00	0.9673 ± 0.0014
PBAT + 12% FORSUD	152 ± 3	0.67 ± 0.00	0.9619 ± 0.0026
PBAT + 14% FORSUD	167 ± 5	0.65 ± 0.00	0.9761 ± 0.0044
PBAT + 16% FORSUD	106 ± 3	0.70 ± 0.00	0.9598 ± 0.0029
PBAT + 10% BMPz	64 ± 12	0.73 ± 0.03	0.9573 ± 0.0037
PBAT + 12% BMPz	44 ± 1	0.77 ± 0.00	0.9232 ± 0.0052
PBAT + 14% BMPz	33 ± 3	0.79 ± 0.01	0.9425 ± 0.0050
PBAT + 16% BMPz	33 ± 2	0.80 ± 0.01	0.8718 ± 0.0556

^a^ Mean values and standard deviations for K, m, and R^2^ values originate from two replications.

**Table 4:** Tensile properties of single- and three-layer films.

Formulation	Tensile Modulus (MPa)	Maximum Tensile Stress (MPa)	Elongation at Break (%)
Neat PBAT ^a^	136 ^b^	35	710
PBAT + 10% FORSUD	33.2 ± 3.2	6.2 ± 0.5	681 ± 19
PBAT + 12% FORSUD	34.5 ± 2.1	5.1 ± 0.5	605 ± 46
PBAT + 14% FORSUD	32.7 ± 5.7	4.3 ± 0.4	473 ± 20
PBAT + 16% FORSUD	30.1 ± 4.1	3.8 ± 0.2	398 ± 25
PBAT + 10% BMPz	34.8 ± 4.4	6.8 ± 0.5	395 ± 76
PBAT + 12% BMPz	57.4 ± 8.7	5.8 ± 0.4	355 ± 88
PBAT + 14% BMPz	51.1 ± 12.7	5.0 ± 0.2	323 ± 50
PBAT + 16% BMPz	51.9 ± 10.0	4.7 ± 0.4	306 ± 26
Three-layer film containing 0% FORSUD BPs ^c^	n.d.	25.1	520
Three-layer film containing 1.0% FORSUD BPs ^d^	n.d.	24.9	550
Three-layer film containing 1.5% FORSUD BPs ^e^	n.d.	23.9	570

^a^ From the technical datasheet (for films with 50 µm thickness); percent values refer to the total mass of the sample. ^b^ Data from reference [14]. ^c^ 15.5 µm thickness. ^d^ 21.7 µm thickness. ^e^ 20.2 µm thickness. n.d., not determined.

## Data Availability

The data that support the findings of this study are available from the corresponding author upon reasonable request.

## Abbreviations

Biopolymers (BPs); Municipal bio-waste anaerobic digestate (FORSUD); Post-harvest tomato plants (BMPz); Poly(Butylene Adipate-Co-Terephthalate) (PBAT).
